# Histological transformation in duodenal-type follicular lymphoma: a case report and review of the literature

**Published:** 2019-05-21

**Authors:** Tomohiko Tanigawa, Ryohei Abe, Jun Kato, Naoki Hosoe, Haruhiko Ogata, Kaori Kameyama, Shinichiro Okamoto, Takehiko Mori

**Affiliations:** ^1^ Division of Hematology, Department of Medicine, Keio University School of Medicine, Tokyo, Japan; ^2^ Center for Diagnostic and Therapeutic Endoscopy, Keio University Hospital, Tokyo, Japan; ^3^ Department of Diagnostic Pathology, Keio University Hospital, Tokyo, Japan

**Keywords:** duodenal-type follicular lymphoma, histological transformation, duodenum, chemotherapy, prognosis

## Abstract

Duodenal-type follicular lymphoma (DFL) is a rare variant of follicular lymphoma (FL) characterized by distinctive clinical features such as localization and favorable prognosis. We herein report a case of DFL in which histological transformation into diffuse large B-cell lymphoma developed 7 years after diagnosis. The transformed lymphoma was refractory to chemotherapy, and the patient passed away due to disease progression. To date, there have been only a limited number of reported cases of histological transformation of DFL, and the clinical outcomes of those cases except our present case have been favorable, with good responses to chemotherapy. Although the histological transformation of DFL is a rare event, the clinical course of the present case suggested that it would be a fatal event and underscore the importance of the life-long management of DFL. Further accumulation of cases is required to elucidate its incidence, characteristics, and prognosis.

## INTRODUCTION

Follicular lymphoma (FL) is a common type of indolent B-cell lymphoma. FL could primarily or secondarily involve extranodal sites, including gastrointestinal tracts. FL accounts for 1-4% of primary non-Hodgkin lymphoma of the gastrointestinal tract [1]. In particular, FL involving the duodenum has been recognized as a unique disease entity, ‘duodenal-type follicular lymphoma’ (DFL) [2]. Several investigators have consistently reported that DFL, which is predominantly confined to the second portion of the duodenum, could also involve the distal small intestine and could present a notably indolent course [3-6]. Histological transformation is an important event in FL and occurs at a rate of 2 to 3% per year; it often has a poor prognosis because of rapid progression and refractoriness to chemotherapy [7]. However, there have been only sporadic reported cases of histological transformation of DFL, and the prognoses of those cases were reported to be favorable, unlike those of FL in general. We here report a case of DFL transforming into diffuse large B-cell lymphoma more than 7 years after diagnosis, whose clinical course was fatal after histological transformation. In addition, previously reported cases of histological transformation of DFL were extensively searched and reviewed.

## CASE REPORT

**Figure 1:**
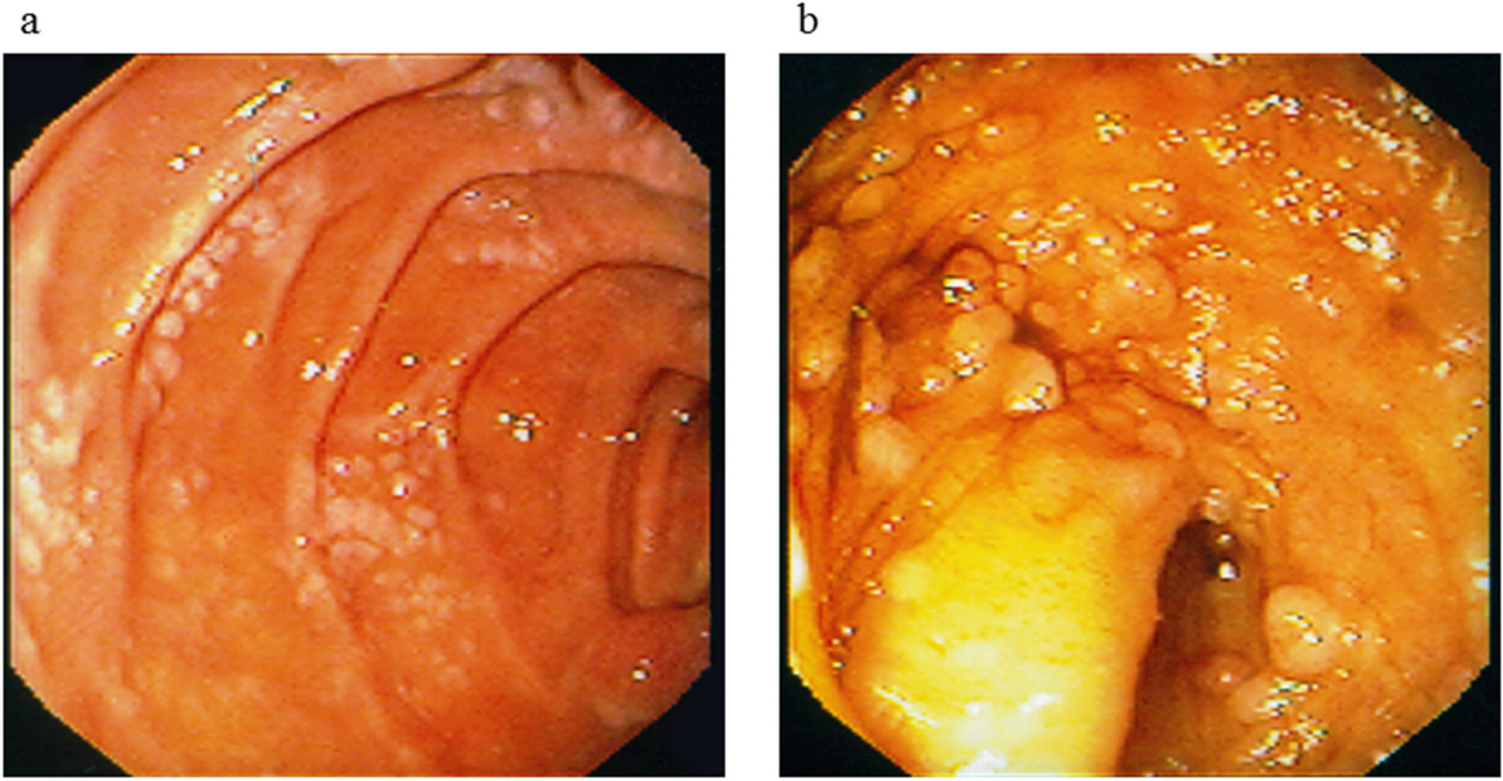
Endoscopic findings at initial presentation. Multiple whitish nodules or plaques were detected at **(a)** the descending portion of the duodenum and **(b)** the terminal ileum.

**Figure 2:**
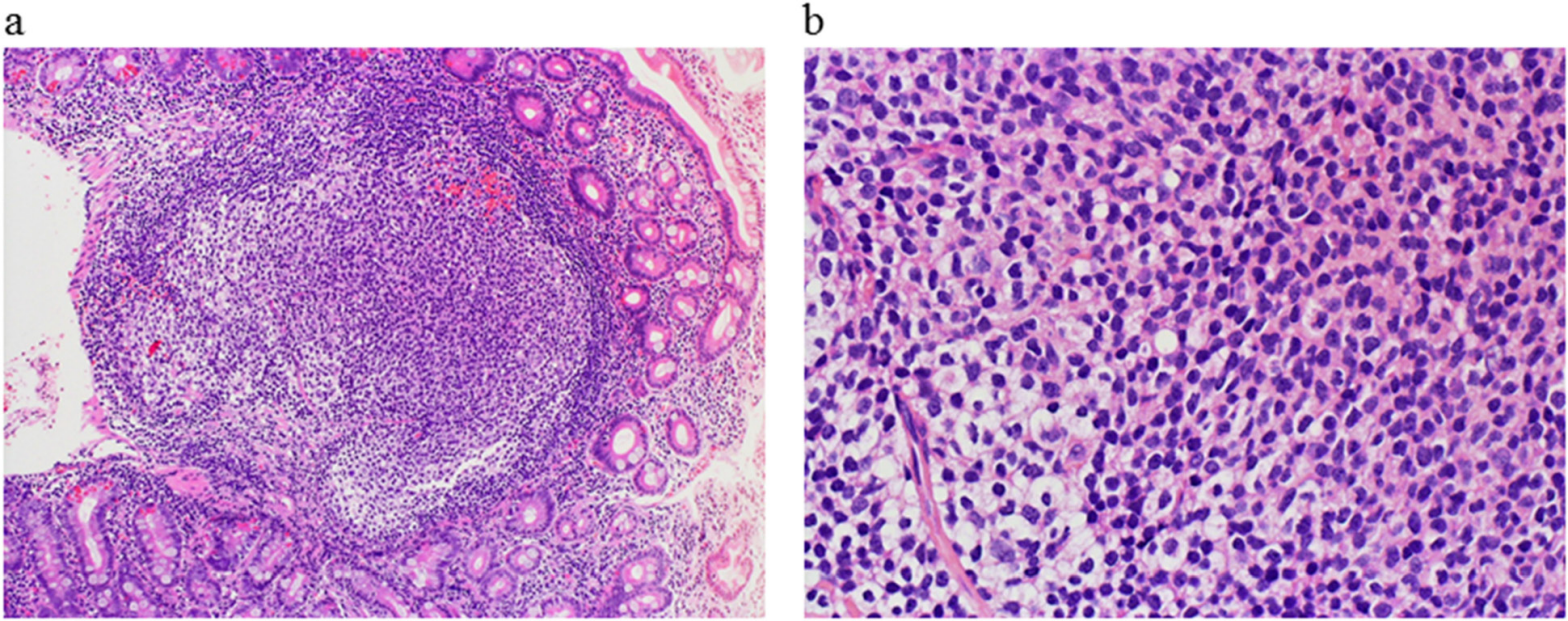
Histological findings of the duodenal tumor at diagnosis. Hematoxylin-eosin staining showed distinct follicles with a monotonous proliferation of small cleaved lymphoid cells (**(a)** low power view; **(b)** high power view).

**Figure 3:**
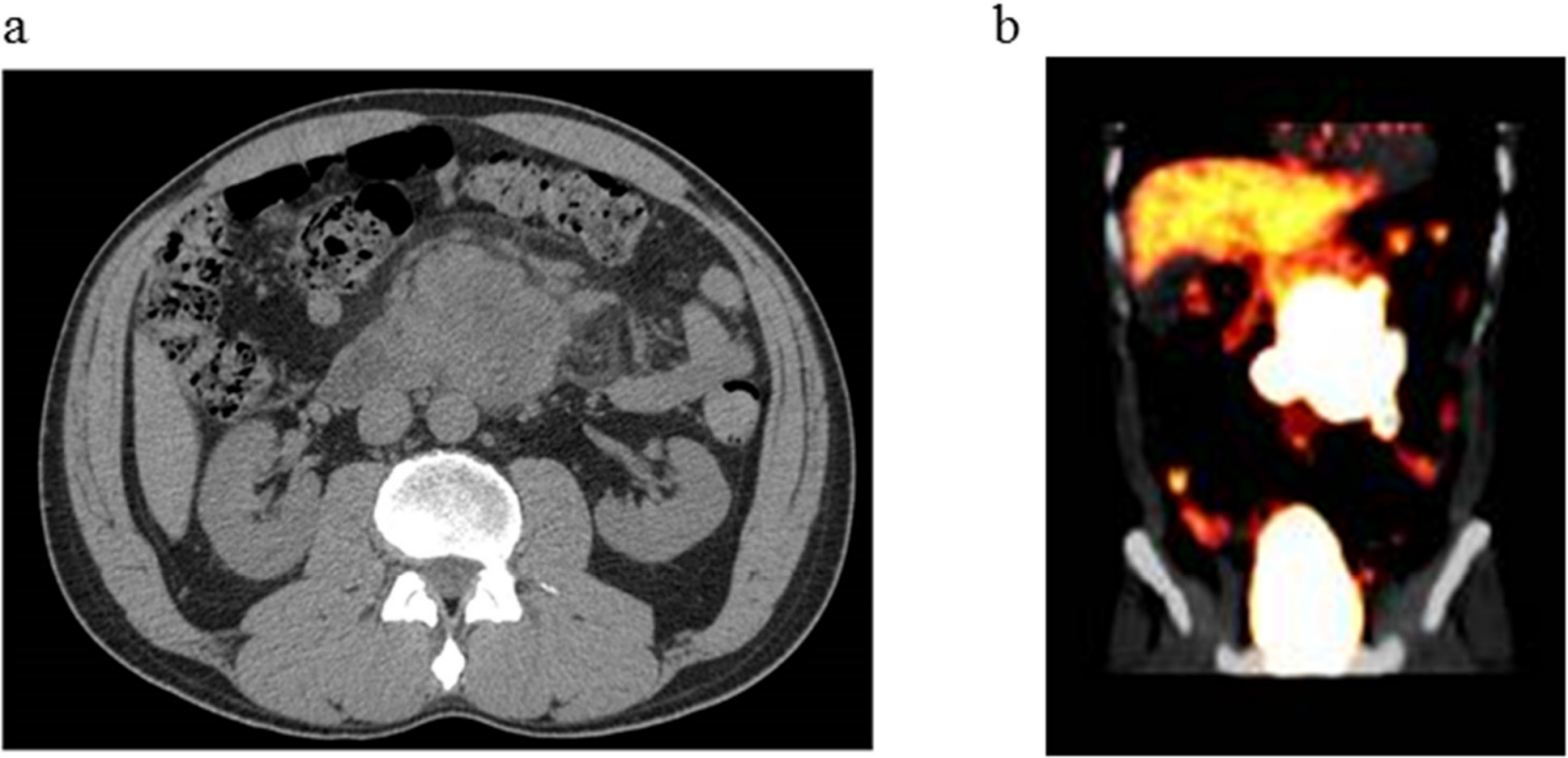
Radiological images of abdominal tumor. **(a)** Computed tomography revealed a 6 cm tumor at the duodenum. **(b)** Positron emission tomography showed a significant uptake at the tumor.

**Figure 4:**
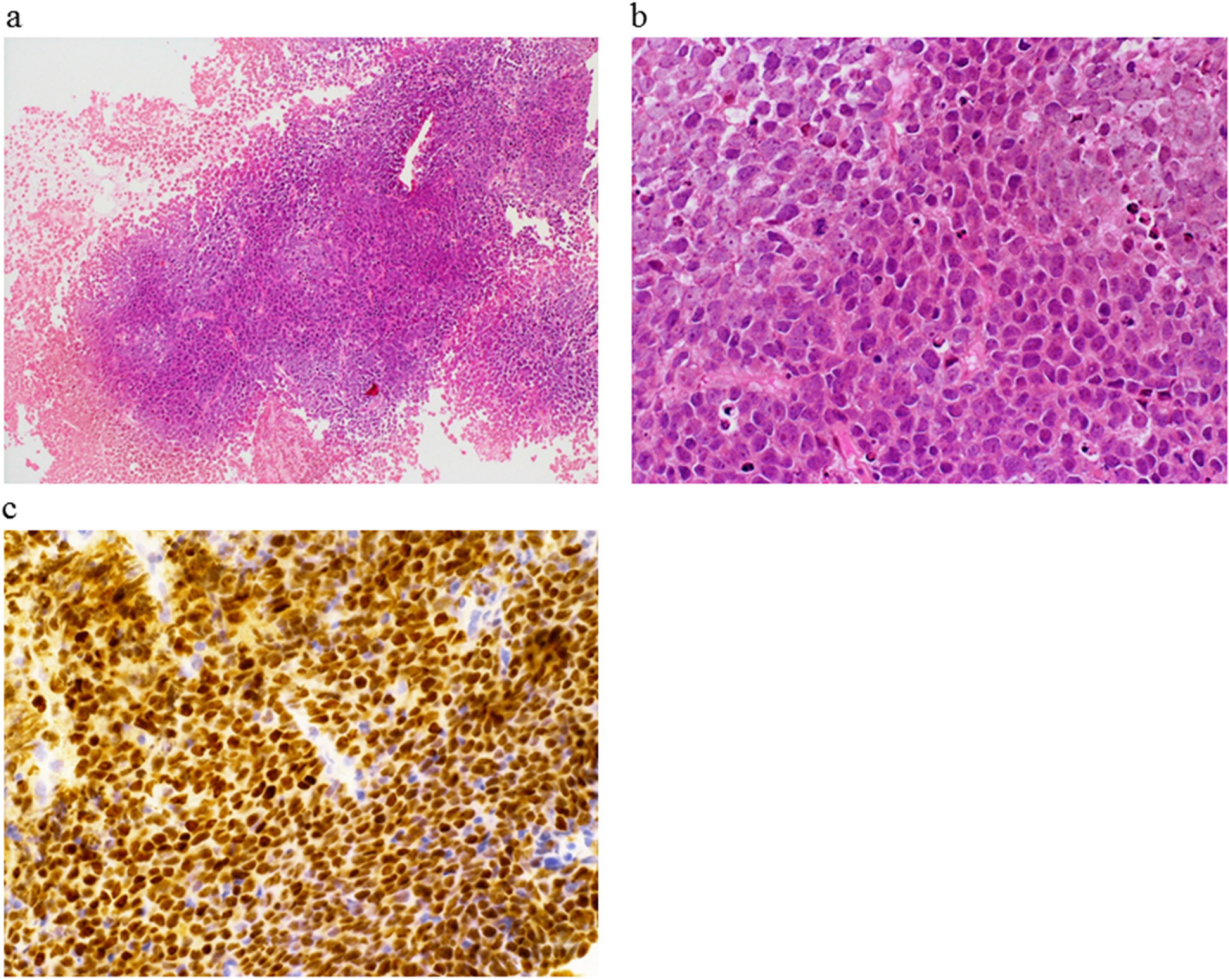
Histological findings of the abdominal tumor at histological transformation. Hematoxylin-eosin staining showed diffuse monotonous proliferation of large lymphoid cells (**(a)** low power view; **(b)** high power view). **(c)** These cells were positive for bcl6 by immunohistochemical staining.

A 52-year-old man underwent gastroduodenoscopy for the examination of mild abdominal fullness, revealing multiple whitish nodules or plaques at the second portion of the duodenum (Figure 1a). A histological diagnosis of grade 1 follicular lymphoma was made on the basis of the findings of distinct follicular-pattern proliferation of monotonous atypical small lymphoid cells (Figure 2a, 2b) that were positive for CD20, CD10, and bcl2. IGH- BCL2 was positive by fluorescence *in situ* hybridization (FISH) analysis. A colonoscopy identified similar lesions at the terminal ileum, which was also histologically confirmed as follicular lymphoma (Figure 1b). Computed tomography (CT), along with bone marrow and cerebrospinal fluid examinations, failed to detect other nodal or extranodal lesions. Serum LDH value was within a normal range. On the basis of those findings, DFL at stage I of the Lugano International Conference Classification was diagnosed. A ‘watch and wait’ policy was chosen, and no treatment was given. A follow-up physical examination and blood tests were given every 2 to 3 months, and CT and endoscopic examinations were performed every 1 to 2 years. However, the patient started complaining of abdominal fullness and upper abdominal pain 7.6 years after the diagnosis was made when he was 60 years old. Gastroduodenoscopy and CT had been performed as routine follow-up 1 and 5 months before the onset, respectively; no significant changes had been documented. However, CT revealed a 6 cm tumor at the duodenum (Figure 3a) and swelling of multiple lymph nodes in the abdominal cavity with significant uptake by positron emission tomography (PET, Figure 3b). PET also detected lesions at the thoracic and lumbar vertebrae. Histological findings of the sample obtained from the abdominal tumor by CT-guided biopsy showed the diffuse proliferation of large atypical lymphoid cells, which were positive for CD20, CD10, bcl2, bcl6, and negative for CD3 and Cyclin D1 (Figure 4). Both IGH-BCL2 and MYC rearrangements were positive by FISH analysis. Follicular component was no longer identified. On the basis of these findings, histological transformation of DFL to diffuse large B-cell lymphoma of germinal center B-cell subtype was diagnosed. The International Prognostic Index was determined as high risk (age, 60; LDH, high (431 U/L); performance status, 1; clinical stage, IV; extranodal sites; two). In the clinical course, serum soluble interleukin-2 receptor was increased to 2026 U/mL. Neither R-CHOP (rituximab, cyclophosphamide, doxorubicin, vincristine, and prednisolone) nor R-ESHAP (rituximab, etoposide, cytarabine, cisplatin, and methylprednisolone) chemotherapy was effective, and the disease progressed. For symptom relief, local field radiotherapy was given, but the response was poor. He succumbed due to disease progression 7 months after the histological transformation and 8 years after the diagnosis of DFL.

### Review of the literature on histological transformation of DFL

Our English-language literature search identified only 7 additional cases of histological transformation of DFL [3, 6, 8-12]. There was another case in a recent report, but we excluded it because of the possibility of systemic FL involving the duodenum [13]. The characteristics of those cases are summarized in Table 1. The median age was 49 years (range, 44-73) at the diagnosis of DFL. The histological grade of FL at diagnosis was 1 in most of the cases, and the clinical stage was I in all. In addition to the duodenum, 2 patients had lesions at the ileum. All but one case (Case 2) had been managed with watch and wait policy without receiving systemic chemotherapy. The time from the diagnosis of DFL to histological transformation ranged from the time of diagnosis to 7 years after diagnosis. In two patients, histological transformation was confirmed at the time of diagnosis of DFL (Cases 6 and 7). Except for our present case, the outcomes after the histological transformation of DFL were favorable, with good responses to CHOP-based chemotherapies.

**Table 1:** Characteristics of reported and present cases of histological transformation from duodenal-type follicular lymphoma

Case	Age (years)	Sex	Histological grade of FL	Lugano stage at diagnosis	Lesions other than duodenum	Treatment for FL	Time from diagnosis to transformation	Treatment for transformed lymphoma	Outcome
1 [3]	N.D.	N.D.	N.D.	N.D.	N.D.	N.D.	4 months	N.D.	N.D.
2 [6]	N.D.	N.D.	N.D.	I	N.D.	Radiation	4.7 months	R-CHOP, radiation	Remission
3 [8]	71	Male	1	I	Ileum	-	5.5-6 years	R-CHOP	Remission
4 [9]	46	Female	N.D.	I	None	-	7 years	R-CHOP	Remission
5 [10]	73	Male	1	I	None	-	62 months	THP-COP	Response
6 [11]	45	Male	1	I	None	-	At diagnosis	R-CHOP	Remission
7 [12]	44	Female	2	I	None	-	At diagnosis	Chemotherapy	Remission
Present case	52	Male	1	I	Ileum	-	7.6 years	R-CHOP, R-ESHAP, radiation	Progression/Death

FL, follicular lymphoma; N.D., not described; R-CHOP, rituximab, cyclophosphamide, doxorubicin, vincristine, prednisolone; THP-COP, pirarubicin, cyclophosphamide, doxorubicin, vincristine, prednisolone; R-ESHAP, rituximab, etoposide, cytarabine, cisplatin, methylprednisolone. Parentheses indicate reference numbers.

## DISCUSSION

Our case of DFL progressed to a histological transformation after a 7-year watch-and-wait follow-up. Histological transformation is a well-recognized feature of FL, and its prognosis is dismal mainly because of the refractoriness to chemotherapy [7]. In contrast, even in the 2 largest studies with long-term follow-up (median 40 and 77 months), including 60 and 100 patients with DFL, no definite case of histological transformation has been documented [4, 5]. Because of the very low incidence of histological transformation, its characteristics and outcomes in the setting of DFL have not been elucidated.

We identified 7 additional reported cases of histological transformation of DFL (Table 1). The patients were middle-aged or elderly, and it was notable that the time from the diagnosis of DFL to histological transformation ranged widely, from the time of diagnosis to 7 years after diagnosis. These findings suggest that histological transformation would be an important event requiring life-long follow-up and that histological transformation at such an early phase of the disease does occur. Involvement of the distal small intestine other than the duodenum was observed only in 2 of the 6 cases with available information. Therefore, at present, it is unlikely that the involvement of the distal small intestine would be predictive of future histological transformation of DFL. All but our case responded to chemotherapy, indicating that our case was the first reported exceptional one to show a fatal outcome of histological transformation from DFL.

The treatment approach for newly diagnosed DFL is still controversial and has not been clearly established. The ‘watch and wait’ approach would be a major option and is generally recommended in the setting of FL [5, 13, 14]. One recent study compared the outcome of management by the ‘watch and wait’ approach in intestinal FL, mostly DFL, with that of rituximab-combined chemotherapy, which revealed a comparable favorable outcome between the groups [14]. However, 4 of 15 patients in the ‘watch and wait’ group experienced disease progression, while 1 in 14 in the chemotherapy group did so. In our literature review, none of the patients with histological transformation had received initial systemic chemotherapy for DFL. However, because of the limited duration and number of evaluated subjects so far, a large-scale study with a longer follow-up is required to evaluate the impact of initial treatment for DFL on future disease progression and histological transformation.

In conclusion, although the incidence is considerably low, physicians should recognize that histological transformation could occur in DFL not only during long-term follow-up but also shortly after diagnosis. An accumulation of cases of histological transformation from DFL is eagerly warranted to further clarify its characteristics and prognosis.

## Abbreviations

DFL: duodenal-type follicular lymphoma,
FL: follicular lymphoma,
CT: computed tomography,
PET: positron emission tomography,
R-CHOP: rituximab, cyclophosphamide, doxorubicin, vincristine, and prednisolone,
R-ESHAP: rituximab, etoposide, cytarabine, cisplatin, and methylprednisolone
